# Supplementary material for: WordCommentsAnalyzer: A windows software tool for qualitative research
Source: F1000Res. 2018 Sep 4;7:536. Originally published 2018 May 3. [Version 2] doi: 10.12688/f1000research.14819.2 (PMC6137411; doi:10.12688/f1000research.14819.2)
Supplement: Supplementary file 3 [file f1000research-7-17614-s0002.tgz › b2a79496-4ada-47c4-9682-a99ce867836d.zip!/Eight Iranian Health Journals/Archives of Rehabilitation_24.docx]

© Archives of Rehabilitation. This work is licensed under CC BY-NC 4.0 (https://creativecommons.org/licenses/by-nc/4.0/).

Volume & Issue: دوره 13 -

Title: مروری بر هندلینگ در کودکان فلج مغزی: از ایده تا عمل

http://rehabilitationj.uswr.ac.ir/article-1-1076-fa.html

امروزه تفسیرهای متعددی در مورد مفهوم، نحوه اجرا، آموزش و تکنیک‌های هندلینگ وجود دارد و بالطبع تعارضات و تفاوت‌هایی هم در انتقال آن توسط درمانگران و متخصصین با ایده‌های مختلف به والدین و مراقبین ایجاد شده و جایگاه هندلینگ در اکوپیشن‌های مرتبط با کاردرمانی نیز دقیقاً مشخص نشده است. لذا هدف از این مطالعه، مروری بر هندلینگ در کودکان فلج مغزی می‌باشد. این پژوهش، یک مطالعه مروری دو مرحله‌ای، در مورد مفهوم و جایگاه هندلینگ در کاردرمانی است که از بانکهای اطلاعاتی داخلی و خارجی بین سال‌های ۱۹۹۰ تا ۲۰۱۲ برای جمع‌آوری اطلاعات استفاده شد. از ۲۶۵۷ مقاله بدست آمده، ۵۰ مقاله کامل مرتبط با موضوع مورد مطالعه قرار گرفتند. طبق مقالات بررسی شده و مستندات، تعاریف متعدد و متفاوتی در مورد هندلینگ وجود دارد. ولی به‌نظر می‌رسد هندلینگ، نوعی مراقبت ویژه از کودکان فلج مغزی است که در حیطه عملکرد کاری و در زمینه‌های مراقبت شخصی، تحرک و عملکرد اجتماعی انجام می‌شود که نه تنها در سلامت کودک، بلکه در ارتقاء سطح سلامت والدین هم نقش اساسی دارد. تمامی اجزاء هندلینگ براساس (ICF-CY) در بخش فعالیت و مشارکت و در زیر بخش زندگی خانوادگی و در حیطه‌های مراقبت از دیگران و پرورش کودک قرار می‌گیرند. علیرغم اینکه هندلینگ جزء حیطه‌های اصلی کاردرمانی است، ولی هنوز تعریف دقیق و جامعی در مورد مفهوم هندلینگ وجود ندارد و فعالیت‌های مرتبط با هندلینگ و جایگاه آن در کاردرمانی تبیین نشده است.

Title: بررسی شکایت از بیماری‌های گوش خارجی و گوش میانی در کودکان کم‌شنوای حسی‌-عصبی و ارتباط آن با شکایت از سمعک

http://rehabilitationj.uswr.ac.ir/article-1-1162-fa.html

هدف: کم‌شنوایی‌های حسی‌عصبی در حال حاضر قابل درمان نمی‌باشند و در بهترین شرایط مطلوب‌ترین روش، استفاده از وسایل کمک شنوایی مناسب و توانبخشی شنوایی است. هدف این مقاله گزارش بررسی شکایت از گوش و سمعک و نیز بررسی ارتباط بین این دو می‌باشد تا توجه درمانگران و اعضای تیم توانبخشی شنوایی به این مسئله تأثیر‌گذار بر فرآیند توانبخشی بیشتر معطوف گردد. روش بررسی: این مطالعه توصیفی تحلیلی بوده و به روش مقطعی بر روی ۹۵ کودک کم‌شنوای حسی‌عصبی در دسترس بین سنین ۱۸-۷ سال صورت گرفته است. هر کودک توسط متخصص گوش، گلو و بینی معاینه می‌گردید و ارزیابی گوش میانی توسط ادیومتری امپدانس صورت می‌گرفت و ارتباط آنها با آزمون کای دو بررسی می‌شد. یافته‌ها: ٪۳۸ کودکانی که از سمعک خود شکایت داشتند، دچار التهاب گوش خارجی بودند. ارتباط معنی‌داری بین شکایت از سمعک و التهاب گوش خارجی وجود داشت P=۰/۰۰۲). ۳۳٪) کودکانی که از گوش خود شکایت داشتند دارای تمپانوگرام غیرطبیعی بودند. ارتباط معنی‌داری میان این دو دیده شد (P=۰/۰۰۵). نتیجه‌گیری: عفونت‌های گوش خارجی و گوش میانی در کودکان کم‌شنوای حسی‌عصبی مانند سایر کودکان شنوا شایع بوده و یکی از علل مهم شکایت از سمعک است که اغلب ناشناخته می‌ماند؛ به‌همین دلیل معاینات دوره‌ای این کودکان توسط متخصص گوش، گلو و بینی و بررسی گوش میانی به کمک امپدانس ادیومتری ضروری به نظر می‌رسد .

Title: تعیین روایی و پایایی تکرار آزمون نسخه والدین و بررسی پایایی بین نسخه درمانگران و نسخه والدین سیستم طبقه‌بندی عملکرد حرکتی درشت در کودکان فلج مغزی

http://rehabilitationj.uswr.ac.ir/article-1-1097-fa.html

هدف: تعیین روایی و پایایی تکرار آزمون نسخه والدین و هم‌چنین بررسی پایایی بین نسخه درمانگران و نسخه والدین سیستم طبقه‌بندی عملکرد حرکتی درشت (GMFCS) در کودکان فلج مغزی هدف این تحقیق می‌باشد. روش‌بررسی: در این مطالعه کاربردی و اعتبارسنجی ۹۰کودک فلج مغزی ۱۲-۲ سال (۳۳ دختر، ۵۷ پسر) با میانگین سنی ۶٫۶ سال با انواع مختلف فلج مغزی مورد بررسی قرار گرفتند. جهت تعیین روایی و پایایی تکرار نسخه والدین و هم‌چنین پایایی بین نسخه والدین و نسخه درمانگران سیستم طبقه‌بندی عملکرد حرکتی درشت، بعد از انجام مراحل ترجمه و بازترجمه، روایی صوری و محتوایی، پایایی تکرار آزمون نسخه والدین و در نهایت پایایی بین نسخه والدین و نسخه درمانگران سیستم طبقه‌بندی عملکرد حرکتی درشت، با استفاده از ضریب همبستگی درون خوشه‌ای (ICC) و ضریب کاپای وزنی مورد ارزیابی قرار گرفت. یافته‌ها:.ضریب همبستگی درون خوشه‌ای والدین (ICC=۰/۹۲) نشان‌دهنده میزان توافق عالی بوده و ضریب کاپای وزنی نیز ۰/۶۱ بوده که نشان‌دهنده میزان توافق خوب بود. پایایی بین ارزیابان نیز عالی بود. ضریب همبستگی درون خوشه‌ای بین نسخه والدین و نسخه درمانگران (ICC=۰/۸۸) به دست آمد و ضریب کاپای وزنی ۰/۶۸ بوده که به ترتیب نشان‌دهنده میزان توافق عالی و خوب می‌باشد. نتیجه‌گیری: نسخه والدین مقیاس GMFCS از روایی و پایایی تکرار آزمون خوبی در میان والدین برخوردار است و هم‌چنین پایایی بین نسخه والدین و درمانگران خوب می‌باشد و می‌توان از آن به عنوان ابزاری مفید و کاربردی در ارزیابی میزان عملکرد حرکتی درشت کودکان فلج مغزی استفاده نمود.

Title: بررسی و مقایسه تغییرات عملکرد حرکتی درشت کودکان فلج‌مغزی براساس شدت درگیری آنها

http://rehabilitationj.uswr.ac.ir/article-1-1133-fa.html

هدف: مشخص کردن ارتباط بین کارکرد حرکتی درشت کودکان مبتلا به فلج‌مغزی با سطح عملکرد حرکتی درشتی که در آن قرار دارند و نیز بررسی و مقایسه تغییرات عملکرد حرکتی درشت کودکان فلج‌مغزی براساس شدت درگیری آن‌ها هدف این تحقیق بوده است. روش بررسی: در این مطالعه مداخله‌ای طولی ۳۰ کودک مبتلا به فلج‌مغزی ۴ تا ۱۲ ساله از کلینیک‌های توانبخشی تهران مطابق معیار‌های تحقیق انتخاب شدند و به صورت غیرتصادفی و براساس سامانه عملکرد حرکتی درشت به سه گروه خفیف، متوسط و شدید تقسیم شدند. ابزار جمع‌آوری داده سامانه طبقه‌بندی‌ عملکرد حرکتی درشت و مقیاس کارکرد حرکتی درشت بود. داده‌ها قبل و بعد از انجام مداخله توسط محقق جمع‌آوری شد. مداخله شامل ۲ جلسه کاردرمانی معمول در هر هفته به مدت سه ماه بود. برای تحلیل داده‌ها از آزمون‌های ضریب همبستگی اسپیرمن، کروسکال والیس، ویلکاکسون و من‌ویتنی استفاده شد. یافته‌ها: بین سطح عملکرد حرکتی درشت کودکان مبتلا به فلج‌مغزی و کارکرد حرکتی درشت آنها در پیش‌آزمون و پس‌آزمون رابطه آماری معنادار و منفی وجود داشت. در پیش‌آزمون میانگین نمره کارکرد حرکتی درشت در گروه خفیف به صورت معناداری بالاتر از متوسط و در گروه متوسط بالاتر از شدید بود (۰/۰۰۱=P)، اما پس از مداخله در میانگین کارکرد حرکتی درشت گروه متوسط و خفیف تفاوت معناداری مشاهده نشد، در حالی که بین گروه متوسط و شدید تفاوت همچنان معنادار بود (۰/۰۰۱=P). بیشترین میزان تغییرات در کارکرد حرکتی درشت متعلق به سطوح ۳ و ۴ بوده است (۰/۰۰۱=P). نتیجه‌گیری: کودکانی که سطح عملکرد حرکتی درشت آن‌ها ۳ و ۴ بود، بیشترین تغییرات کارکرد حرکتی درشت را متعاقب بر خورداری از مداخلات کاردرمانی نشان دادند.

Title: اثربخشی برنامه گروهی والدگری مثبت بر نشانه‌های اختلال بیش‌فعالی/نارسایی توجه در کودکان

http://rehabilitationj.uswr.ac.ir/article-1-1094-fa.html

هدف: اختلال بیش‌فعالی/نارسایی توجه یکی از شایع‌ترین اختلالات روانپزشکی دوران کودکی می‌باشد. یکی از درمان‌های مؤثر در این اختلال که در ترکیب با دارودرمانی به کار می‌رود آموزش والدگری مثبت است. هدف مطالعه حاضر، تعیین اثربخشی برنامه گروهی والدگری مثبت بر نشانه‌های اختلال بیش‌فعالی/نارسایی توجه در کودکان ۴ تا ۱۰ سال بود. روش بررسی: در این مطالعه پیش تجربی، از طرح پیش‌آزمون و پس‌آزمون تک گروهی استفاده شد و مادران ۲۶ کودک با اختلال بیش‌فعالی/نارسایی توجه، مراجعه کننده به مراکز اخوان و رفیده به شیوه هدفمند و در دسترس انتخاب شدند و در ۸ جلسه آموزشی برنامه گروهی والدگری مثبت (یک جلسه ۲ ساعته در هر هفته) شرکت کردند. مادران به فرم کوتاه و تجدید نظرشده مقیاس درجه‌بندی کانرز والدین، قبل و بعد از شرکت در جلسات آموزشی پاسخ دادند. داده‌ها با استفاده از آزمون تحلیل واریانس یک متغیری با اندازه‌گیری مکرر و آزمون تعقیبی بونفرونی تحلیل شدند. یافته‌ها: نتایج آزمون تحلیل واریانس با اندازه‌گیری مکرر نشان داد که آموزش برنامه گروهی والدگری مثبت به مادران به ترتیب منجر به کاهش میانگین شاخص بیش‌فعالی/نارسایی توجه، بیش‌فعالی، مخالفت‌جویی، و مشکلات شناختی و توجه شده است. نتایج آزمون تعقیبی بونفرونی بیانگر آن بود که تفاوت بین زوج میانگین‌های بیش‌فعالی با مخالفت‌جویی و مشکلات شناختی و توجه، شاخص بیش‌فعالی/نارسایی توجه با مخالفت‌جویی، مشکلات شناختی و توجه، و بیش‌فعالی معنادار (۰/۰۵ نتیجه‌گیری: آموزش گروهی برنامه والدگری مثبت موجب بهبود نشانه‌های اختلال در کودکان با اختلال بیش‌فعالی/نارسایی توجه می‌شود و به عنوان روش ترکیبی با دارو درمانی به نتایج مثبت می‌انجامد.

Title: مقایسه آزمون‌ غربالگری دنور ۲ و پرسش‌نامه‌های سنین و مراحل در بخش تکامل حرکتی کودکان ۶۰-۴ ماهه شهر تهران

http://rehabilitationj.uswr.ac.ir/article-1-1131-fa.html

هدف: این مطالعه به منظور بررسی میزان توافق تکامل حرکتی (ظریف و درشت) آزمون‌های غربالگری تکاملی دنور۲ و پرسش‌نامه سنین و مراحل (ASQ) در کودکان ۶۰-۴ ماهه شهر تهران و مقایسه نتایج دو آزمون در بخش تکامل حرکتی و تعیین ضریب توافق دو آزمون صورت گرفت. روش بررسی: ۱۹۷ کودک (۱۰۴ پسر و ۹۳ دختر) ۴ تا ۶۰ ماهه بوسیله DDST-II و ASQ در چهار مرکز بهداشتی‌درمانی در شمال، جنوب، شرق و غرب تهران در این مطالعه تحلیلی و کاربردی مورد بررسی تکامل حرکات درشت و ظریف قرار گرفتند. نمونه‌گیری به روش نمونه‌گیری در دسترس تا تکمیل حجم نمونه انجام شد. اطلاعات مورد تجزیه و تحلیل قرار گرفت و ضریب توافق دو آزمون تعیین شد. یافته‌ها: در بررسی انجام شده با DDST-II و ASQ به ترتیب ٪۷/۱۱ و ٪۵ از کودکان مشکوک به تأخیر تکامل حرکات ظریف و ٪۱۱/۲ و ٪۶/۳ مشکوک به تأخیر تکامل حرکات درشت شناخته شدند. ضریب توافق کاپا بین دو آزمون در حیطه حرکات ظریف ۰/۰۵ و در حیطه حرکات درشت ۰/۲۴ بود. نتیجه‌گیری: در این مطالعه از بررسی تکامل حرکتی کودکان تهرانی با دو ابزار دنور۲ و ASQ، نتایج متفاوتی به دست آمده و ضریب توافق کاپا بین دو آزمون ضعیف است. برای انتخاب یک ابزار غربالگر مناسب حرکتی، نتایج غربالگری باید با یک آزمون استاندارد تشخیصی طلایی مقایسه شوند.

Title: سلامت عمومی، عزت نفس و مهارت‌های اجتماعی خواهران و برادران دانش‌آموزان کم‌توان ذهنی آموزش‌پذیر و عادی

http://rehabilitationj.uswr.ac.ir/article-1-688-fa.html

هدف: هدف از پژوهش حاضر مقایسه سلامت عمومی، عزت نفس و مهارت‌های اجتماعی خواهران و برادران، دانش‌آموزان کم‌توان‌ذهنی آموزش پذیر و عادی در شهر یزد می‌باشد. روش بررسی: این پژوهش یک مطالعه مورد-شاهدی است. ۸۰ نفر از خواهران و برادران، دانش‌آموزان کم‌توان‌ذهنی و ۸۳ نفر از دانش‌آموزان عادی با استفاده از روش نمونه‌گیری خوشه‌ای چندمرحله‌ای به عنوان گروه نمونه انتخاب گردید و پرسش‌نامه سلامت عمومی، عزت نفس رزنبرگ و مهارت‌های اجتماعی متسون در اختیار آنها قرار گرفت. داده ها با استفاده از ضریب همبستگی پ&zwjیرسون و آزمون‌های آماری کولموگروف-اسمیرنوف‌وتی مستقل تحلیل شد. یافته‌ها: نتایج بدست آمده از آزمون t مستقل، تفاوت معناداری بین خواهران دانش‌آموزان کم‌توان‌ذهنی در مهارت‌‌های اجتماعی از جمله: ترس از روابط با همسالان (۰/۰۰۱=P) و رفتار‌های نوع‌دوستانه (۰/۲۴=P) نسبت به خواهران دانش‌آموزان عادی نشان داد و در سایر موارد تفاوت معناداری بین سایر گروه‌ها مشاهده نگردید. نتیجه‌گیری: با توجه به نتایج پژوهش حاضر، ترس از روابط با همسالان و رفتار‌های نوع دوستانه در خواهران دانش‌آموزان کم‌توان‌ذهنی بیشتر از سایر گروه‌ها مشاهده می‌شود. باید به این نکته اشاره کرد که عواملی، ازجمله: تأثیر این کودکان در محیط خانواده، داشتن اطلاعات کافی نسبت به کودکان کم‌توان‌ذهنی و میزان سازگاری کودک کم‌توان‌ذهنی می‌توانند عوامل مهم در ارتباط دختران و پسران با خواهر یا برادر کم‌توان‌ذهنی، سلامت عمومی، عزت نفس و مهارت‌‌های اجتماعی آنها باشد.

Title: ارتباط کم‌خونی ‌مادر در هنگام زایمان با وضعیت تکاملی کودکان ۱۲ ماهه مراجعه‌کننده به مراکز بهداشتی-درمانی وابسته به دانشگاه علوم پزشکی و خدمات بهداشتی-درمانی قزوین سال ۱۳۹۰

http://rehabilitationj.uswr.ac.ir/article-1-1053-fa.html

هدف: به دلیل ضرورت پیگیری‌های بیشتر و متفاوت در کودکانی با اختلالات تکاملی در سال‌های اولیه عمر، تعیین عوامل موثر در دوران بارداری در بروز اختلالات تکامل در هر جامعه‌ای اهمیت دارد. این مطالعه با هدف تعیین همبستگی کم‌خونی مادر هنگام زایمان با وضعیت تکاملی کودکان ۱۲ ماهه انجام گرفت. روش بررسی: تحقیق با طراحی توصیفی همبستگی بر روی ۲۵۰ مادر و کودک ۱۲ ماهه آنان، مراجعه‌کننده به مراکز بهداشتی-درمانی وابسته به دانشگاه علوم پزشکی و خدمات بهداشتی-درمانی قزوین سال ۱۳۹۰ به روش نمونه‌گیری چند مرحله‌ای انجام شد. ابزار گردآوری داده‌ها شامل فرم اطلاعاتی و پرسش‌نامه‌‌های سنین و مراحل بود. داده‌ها با آزمون‌های مجذور کای، تی مستقل، من ویتنی و رگرسیون لجستیک مورد تجزیه و تحلیل آماری قرار گرفت. یافته‌ها: شیوع آنمی‌ فقرآهن در جامعه مورد نظر ۱۲ درصد و شیوع تأخیر تکاملی کودکان ۲۲٫۴ درصد می‌باشد. آنمی‌ همبستگی معناداری با وضعیت تکاملی کودکان و حیطه ارتباطی آن نشان داد (۰/۰۵>P). در مدل لجستیک متغیر میزان هموگلوبین همبستگی معناداری با وضعیت تکاملی کودکان نشان داد (۰/۰۲=P و ۰/۳۱=OR). از کل کودکان ۶۶٫۷ درصد در گروه آنمیک و ۵۵ درصد در گروه غیر آنمیک پسر بودند. ارتباط جنسیت پسر با تأخیر تکاملی مقدار احتمال نزدیک به سطح معناداری داشت (۰/۰۵۵=P و ۰/۵۲=OR). نتیجه‌گیری: به نظر می‌رسد یکی از دلایل تأخیر تکاملی کودکان آنمی می‌باشد. لزوم تحقیقات بیشتر و نیز تاکید بر مصرف آهن در دوران بارداری توصیه می‌گردد.

Title: روایی و پایایی نسخه فارسی پرسش‌نامه کیفیت زندگی کودکان فلج مغزی

http://rehabilitationj.uswr.ac.ir/article-1-1088-fa.html

هدف: هدف از مطالعه حاضر تهیه نسخه فارسی پرسش‌نامه "کیفیت زندگی کودکان فلج مغزی" و بررسی روایی و پایایی آن بود. روش‌بررسی: در این مطالعه مقطعی با روش توصیفی-تحلیلی و اعتبار سنجی طی ۵ مرحله پرسش‌نامه کیفیت زندگی کودکان فلج مغزی به زبان فارسی ترجمه و سپس روایی و پایایی آن بررسی شد. والد ۲۰۰ کودک فلج مغزی ۱۲-۴ ساله از جامعه در دسترس مراجعه‌کننده به ۸ مرکز منتخب توانبخشی و ۳ مدرسه کودکان جسمی-حرکتی نمونه‌گیری شد. داده‌های به دست آمده از نظر پایایی (تکرار پذیری به فاصله ۳ هفته و هم‌خوانی درونی) و روایی [ظاهری، ساختاری با سامانه طبقه‌بندی توانایی دستی (MACS) و سامانه طبقه‌بندی عملکرد حرکتی درشت (GMFCS) و افتراقی آیتم‌ها] مورد بررسی قرار گرفت. یافته‌ها: ضریب همبستگی اسپیرمن بین این پرسش‌نامه و آزمون ام.ای.سی.اس (r=۰/۴۰-۰/۱۳) و آزمون جی.ام.اف.سی.اس  (۰/۳۲-۰/۱۸=r) نشان‌دهنده ارتباط متوسط آنهاست. ٪۷۵ موارد همبستگی نمرات هر یک از سؤالات با نمره خرده‌مقیاس مربوطه معنی‌دار و مثبت بود. این نتایج نمایانگر روایی افتراقی مناسب آیتم‌ها برای این پرسش‌نامه است. آلفای کرونباخ ۰/۸۷-۰/۶۱ و خطای معیار اندازه‌گیری ۱۵/۲۷-۴/۸ و ضریب همبستگی درون رده‌ای ۰/۸۴-۰/۴۷بود که نمایانگر پایایی بالایی برای نسخه فارسی این پرسش‌نامه می‌باشد. نتیجه‌گیری: نسخه فارسی پرسش‌نامه «کیفیت زندگی کودکان فلج مغزی» از پایایی و روایی قابل قبولی در انواع مورد بررسی برخوردار است و بنابراین می‌تواند به عنوان ابزار بالینی و تحقیقاتی مورد استفاده واقع شود.

Title: ساخت و اعتباریابی پرسش‌نامه کیفیت زندگی وابسته به کم‌شنوایی نوجوانان

http://rehabilitationj.uswr.ac.ir/article-1-1099-fa.html

هدف: هدف از انجام پژوهش حاضر ساخت ابزاری معتبر برای سنجش کیفیت زندگی وابسته به کم‌شنوایی برای نوجوانان کم‌شنوا بود . روش بررسی: نسخه اولیه پرسش‌نامه و سیستم پاسخگویی و نمره‌دهی براساس مرور منابع طراحی شد. برای تعیین روایی صوری و محتوایی نظر ۱۴ نفر از صاحب‌نظران کسب شد. برای تعیین وضوح متن و بررسی روایی سازه و پایایی آزمون-بازآزمون پرسش‌نامه توسط نوجوان کم‌شنوای ۱۸-۱۲ سال شاغل به تحصیل در مقاطع راهنمایی و دبیرستان مدارس عادی و استثنایی شهر تهران در سال ۱۳۹۰ تکمیل شد. دانش آموزان مورد نظر براساس نمونه‌گیری طبقه‌بندی شده انتخاب شدند و حجم نمونه به ترتیب برابر ۲۵۵ ،۱۱۷ و ۲۸ نفر بود. اندکس روایی محتوایی به شیوه لاواشه محاسبه شد و برای تعیین روایی سازه تحلیلی عاملی انجام شد. یافته‌ها: پرسش‌نامه در سه محور شامل ۳۸ سوال ۳ تا ۵ گزینه‌ای ساخته شد. نمره کل پرسش‌نامه و نمرات هر یک از سه محور در مقیاسی ۱۰۰-۰ نمره‌ای طراحی شد که در آن نمره بالاتر نشانه کیفیت زندگی بهتر است. روایی صوری و روایی محتوایی (اندکس روایی محتوایی=۰/۷۹) مورد تأیید قرار گرفت. پایایی آزمون-بازآزمون ابزار برای نمره کل برابر با ۰/۸۵ و برای هر یک از سه محور جسمی، عاطفی و اجتماعی به ترتیب برابر ۰/۸۱،۰/۶۸و ۰/۷۸ محاسبه شد. نتیجه‌گیری: پرسش‌نامه کیفیت زندگی وابسته به کم‌شنوایی نوجوانان ابزاری روا و پایا است و می‌تواند در سنجش اثرات کم‌شنوایی بر کیفیت زندگی نوجوانان ایرانی و هم‌چنین ارزیابی پیامد‌‌های ارائه خدمات توانبخشی به ایشان مورد استفاده قرار گیرد.

Title: پدیده ترخیص کودکان کم‌شنوا از خدمت تربیت‌شنوایی از دیدگاه شنوایی‌شناسان

http://rehabilitationj.uswr.ac.ir/article-1-1135-fa.html

هدف: بسیاری از کودکان کم‌شنوا پس از تشخیص، برای دریافت خدمت تربیت‌ شنیداری ارجاع می‌شوند. هدف پژوهش آن بود که پدیده ترخیص از خدمات تربیت‌ شنیداری کودکان کم‌شنوا مورد کنکاش و شناسایی قرار دهد. روش بررسی: در این پژوهش کیفی که در تهران انجام شد، برای جمع‌آوری داده‌ها از شیوه مصاحبه عمیق نیمه‌ساختار یافته با شنوایی‌شناسان باتجربه استفاده شد. برای دست‌یابی به شنوایی‌شناسان با تجربه در ارائه خدمت توانبخشی تربیت ‌شنوایی به کودکان کم‌شنوا ترکیبی از شیوه‌های نمونه‌گیری هدفمند و گلوله برفی به کار برده شد. نمونه‌گیری تا آنجا ادامه یافت که اشباع داده‌ها حاصل شد، و این زمانی بود که تعداد مصاحبه‌شوندگان بالغ بر ۸ نفر شدند. متونی مشتمل بر دست‌نوشته‌هایی که در طول مصاحبه از نکات مهم فراهم شده و پیاده‌سازی متن از نوار ضبط شده مصاحبه‌ها تهیه شد. طی فرآیند کدگذاری-استخراج گویه مفاهیم شکل گرفتند. کد‌ها در گروه‌های بزرگ‌تر تم‌ها و خوشه‌ها دسته‌بندی شدند. یافته‌ها: برخی از مصاحبه‌شوندگان «تکامل توانایی‌های گوش دادن» و «دریافت خدمات تربیت‌ شنیداری» را دو مقوله متمایز از یکدیگر نمی‌پنداشتند. به‌نظر می‌رسد «تمایل و یا محدودیت‌های خانواده» تنها معیار تعریف شده و مشخص مورد اتفاق نظر مشارکت‌کنندگان برای پایان دادن به جلسات درمانی بود. نتایج نشان داد بیشتر مشارکت‌کنندگان بر این باور نبودند که لزوماً می‌بایست برای برنامه ارائه خدمت تربیت‌ شنیداری نقطه پایانی در نظر داشت. هر چند مشارکت‌کنندگانی که باور داشتند که می‌بایست نقطه‌ای را برای ترخیص توانجو از خدمت تربیت‌ شنیداری تعریف کرد، وسایل موجود ارزیابی مهارت‌های شنیداری کودکان را به عنوان وسیله‌ای برای بر مبنای آن بتوان برای پایان دادن به جلسات تربیت‌ شنیداری تصمیم گرفت ناکارآمد می‌دانستند. نتیجه‌گیری: نبود اتفاق نظر در مورد ضروری بودن داشتن برنامه ترخیص از خدمت تربیت‌ شنیداری، عدم کارایی ابزارهای سنجش مهارت‌های ‌شنوایی موجود در ارزیابی توانایی‌ها و تعیین آمادگی توان‌جویان برای ترخیص تنگناهایی است که در مدیریت کارای توانبخشی کودکان شناسایی شده در برنامه کشوری غربال شنوایی نوزادان، وجود دارد.

Title: بررسی اثربخشی آموزش کفایت اجتماعی بر رفتار سازشی دانش‌آموزان کم‌توان‌ ذهنی

http://rehabilitationj.uswr.ac.ir/article-1-1151-fa.html

هدف: هدف این پژوهش بررسی اثربخشی آموزش کفایت اجتماعی بر رفتار سازشی دانش‌آموزان پسر کم‌توان ذهنی شهرستان‌های تهران بود. روش بررسی: پژوهش حاضر از نوع شبه‌آزمایشی با طرح پیش آزمون- پس آزمون با گروه کنترل است. جامعه آماری این پژوهش را دانش‌آموزان پسر کم‌توان ذهنی 12 تا 14 ساله شهرستان‌های تهران تشکیل دادند. برای انتخاب آزمودنی‌ها با استفاده از روش نمونه‌گیری تصادفی خوشه‌ای ابتدا از بین شهرستان های تهران، 2 شهرستان و از هر شهرستان یک مدرسه به صورت تصادفی انتخاب شدند، سپس از هر مدرسه 20 دانش‌آموز انتخاب شد که به طور تصادفی در یکی از دوگروه آزمایش و کنترل جایگزین شدند. گروه‌ آزمایشی، طی 10 جلسه تحت آموزش کفایت اجتماعی قرار گرفت ولی به گروه کنترل آموزشی ارائه نشد. ابزار جمع‌آوری اطلاعات آزمون هوش ریون، مقیاس رفتار سازشی واینلند و برنامه آموزشی کفایت اجتماعی بود. داده‌های به دست آمده با استفاده از تحلیل کوواریانس چند متغیری تحلیل شد. یافته‌ها: نتایج نشان داد که میانگین نمرات رفتار سازشی گروه آزمایش به طور معناداری بیشتر از گروه کنترل بود (P<0/0005). همچنین نمرات رفتار سازشی گروه آزمایش در مهارت‌ ارتباطی، مهارت‌ زندگی و مهارت اجتماعی معنادار بود (P<0/0005)، ولی در مهارت حرکتی معنادار نبود (P<0/269). نتیجه‌گیری: برنامه آموزشی کفایت اجتماعی باعث بهبود رفتار سازشی، مهارت‌ ارتباطی، مهارت‌ زندگی و مهارت اجتماعی دانش‌آموزان کم‌توان ذهنی شد. بنابراین برنامه‌ریزی برای ارائه آموزش کفایت اجتماعی به این دانش‌آموزان اهمیت ویژه‌ای دارد.

Title: تأثیر آموزش مهارت‌های اجتماعی بر مهارت‌های زندگی روزمره دختران با سندرم داون

http://rehabilitationj.uswr.ac.ir/article-1-1077-fa.html

هدف: هدف از پژوهش حاضر، تعیین تأثیر آموزش مهارت‌های اجتماعی بر مهارت‌های زندگی روزمره کودکان سندرم داون بود. روش بررسی: این پژوهش یک مطالعه شبه تجربی بود. ۴۰ دانش آموز با سندرم داون در دامنه سنی ۸ تا ۱۲ سال به صورت در دسترس انتخاب و به طور تصادفی به دو گروه مداخله و کنترل (۲۰ نفر در هر گروه) تقسیم شدند. گروه مداخله به مدت ۱۰ جلسه (هر جلسه یک ساعت) طی یک دوره ۲ ماهه تحت مداخله آموزش مهارت‌های اجتماعی قرار گرفتند. جهت جمع‌آوری داده‌ها ازخرده‌مقیاس مهارت‌های زندگی روزمره واینلند به عنوان پیش‌آزمون، پس‌آزمون و آزمون پیگیری در دو گروه استفاده گردید. جهت تجزیه و تحلیل داده‌ها از آزمون آماری تحلیل واریانس مکرر ترکیبی استفاده شد. یافته‌ها: بین دو گروه از نظر نمرات مهارت‌های زندگی روزمره تفاوت معناداری مشاهده شد. مطالعه پیگیری نیز نشان داد این اثر پس از خاتمه آموزش نیز همچنان حفظ شده است (۰/۰۰۱>P). نتیجه‌گیری: به نظر می‌رسد آموزش مهارت‌های اجتماعی می‌تواند موجب بهبود مهارت‌های زندگی روزمره در کودکان مبتلا به سندرم داون گردد.

Title: تأثیر برنامه مکیدن غیرتغذیه‌ای بر زمان دستیابی به تغذیه دهانی کامل در نوزادان نارس

http://rehabilitationj.uswr.ac.ir/article-1-1090-fa.html

هدف: هدف این مقاله بررسی تأثیر برنامه مکیدن غیرتغذیه‌ای بر زمان دستیابی به تغذیه دهانی کامل و وزن‌گیری نوزادان نارس ۳۲-۲۶ هفته جنینی بستری در واحد مراقبت‌‌های ویژه نوزادان بیمارستان کمالی کرج می‌باشد. روش بررسی: مطالعه از نوع کارآزمایی بالینی می‌باشد. جامعه مورد مطالعه، نوزادان نارس با سن جنینی ۳۲-۲۶ هفته، بستری در واحد مراقبت‌های ویژه نوزادان بیمارستان کمالی کرج می‌باشند. ۲۲ نوزاد به صورت تصادفی در دو گروه "مداخله مکیدن غیر تغذیه‌ای" و "گروه کنترل" قرار گرفتند. به گروه درمانی مکیدن غیرتغذیه‌ای، در طی ۵ دقیقه اول تغذیه لوله‌ای (۳ بار در روز) و به مدت ۱۰ روز تحریک دهانی ارائه گردید. نوزادان گروه کنترل به غیر از خدمات روتین پرستاری، هیچ‌گونه تحریکی را دریافت نمی‌کردند. آزمودنی‌ها از لحاظ سن جنینی در دفعات مختلف تغذیه دهانی و در زمان ترخیص از واحد مراقبت‌‌های ویژه نوزادان و میزان وزن‌گیری در هفته اول، دوم تولد و زمان ترخیص مورد بررسی و مقایسه بین دو گروه قرار گرفتند. یافته‌ها: در گروه مکیدن غیر تغذیه‌ای، میانگین سن جنینی در زمان ترخیص ۰/۶۹±۳۳/۹۷ هفته و در گروه کنترل، میانگین سن جنینی در زمان ترخیص ۱/۳۳±۳۴/۳۲ هفته بوده است که از لحاظ آماری معنادار نمی‌باشد (۰/۴=P). در گروه مکیدن غیر تغذیه‌ای، میانگین وزن زمان ترخیص ۱۳۳/۲۹±۱۶۵۴/۵۴ گرم در گروه کنترل، میانگین وزن زمان ترخیص۹۴/۳۴±۱۴۷۲/۷۲گرم بوده است. وزن زمان ترخیص گروه مکیدن غیر تغذیه‌ای به طور معناداری بیشتر از گروه کنترل است (۰/۰۰۱=P). نتیجه‌گیری: استفاده از مکیدن غیرتغذیه‌ای، به طور موثری وزن‌گیری را افزایش می‌دهد؛ هرچند که به لحاظ آماری اثر معناداری بر معیار‌های تغذیه دهانی نداشت اما به لحاظ بالینی دیده شد که تغذیه دهانی را در نوزادان نارس تسهیل می‌کند و مدت زمان بستری در بیمارستان را کاهش می‌دهد.

Title: مقایسه کارکرد‌های اجرایی در کودکان مبتلا به اوتیسم و عادی و ارتباط آن‌ها با توانایی ریاضیات و خواندن

http://rehabilitationj.uswr.ac.ir/article-1-1126-fa.html

هدف: پژوهش حاضر با هدف مقایسه کارکرد‌های اجرایی در افراد اوتیسم و عادی و ارتباط آن‌ها با توانایی ریاضیات و خواندن انجام شد. روش بررسی: پژوهش حاضر یک مطالعه توصیفی-مقایسه‌ای بود. نمونه مطالعه شامل ۱۳۲ دانش آموز (۱۶ اوتیسم و ۱۱۶ عادی) بود. گروه عادی با روش نمونه‌گیری در دسترس انتخاب شدند. به علت محدود بودن جامعه آماری کودکان اوتیسم، تمام شماری انجام شد و دو گروه در برخی متغیرها همتا شدند. از آزمون‌‌های کولیج و کورنولدی، کی-مت و سطح خواندن به ترتیب برای اندازه‌گیری کارکرد‌های اجرایی، توانایی ریاضیات و خواندن استفاده شد. داده‌ها با استفاده از تحلیل واریانس چند متغیره آنالیز شد. یافته‌ها: کودکان اوتیسم به طور معنادار کارکرد‌های اجرایی ضعیف‌تری نسبت به کودکان عادی داشتند. هم‌چنین نتایج تحلیل رگرسیون چندگانه نشان داد که عوامل‌های سازماندهی و تصمیم‌گیری-برنامه‌ریزی در پیش‌بینی توانایی ریاضیات و بازداری و حافظه فعال در پیش‌بینی سطح خواندن سهم بیشتری دارند. نتیجه‌گیری: براساس این یافته‌ها کارکرد‌های اجرایی در کودکان اوتیسم مختل هستند و اختلال در آن‌ها با ضعف در ریاضیات و خواندن رابطه دارد.

Title: بررسی تأثیر آموزش مهارت‌های تمرین توپ بر رفتار تطابقی کودکان مبتلا به اوتیسم با عملکرد بالا

http://rehabilitationj.uswr.ac.ir/article-1-1139-fa.html

هدف: هدف از انجام این پژوهش بررسی تاثیر آموزش مهارت های تمرین توپ بر رفتار تطابقی کودکان 4 تا 7 ساله مبتلا به اوتیسم با عملکرد بالا می باشد. روش بررسی: 14 کودک (12پسر و 2 دختر) با میانگین سنی 43/7±3/64 ماه در این مطالعه شرکت داشتند. از کلیه شرکت کنندگان در سه مرحله آزمون های رفتار تطابقی واینلند و ارزیابی درمان اوتیسم بعمل آمد. این پژوهش در دو فاز طراحی گردید. در فاز اول بعد از ارزیابی آغازین شرکت کنندگان به درمان رایج خود ادامه دادند. بعد از مدت 1 ماه ارزیابی اولیه انجام گردید. فاز دوم بلافاصله با شروع مداخلات درمانی (هفته ایی 3 جلسه 45 دقیقه ایی و مجموعا 10 جلسه در یک ماه) آغاز گردید. بعد از اتمام جلسات درمانی ارزیابی ثانویه انجام شد. در فاز دوم شرکت کنندگان به درمان رایج ادامه دادند. یافته‌ها: بررسی تفاضل میانگین نمرات آزمون ارزیابی درمان اوتیسم به روش مقایسات بن فرونی نشان داد که تغییرات حاصل در هر دو فاز پژوهش اثر آماری معناداری بر روی برخی داده ها داشتند. البته افزایش قابل ملاحظه ایی در میانگین نمرات بعد از انجام مداخله و در فاز دوم مشاهده شد. در خرده آزمون های ارتباط کلامی، اجتماعی شدن و فعالیت های زندگی روزمره آزمون واینلند، فاز اول درمان تاثیر آماری معناداری روی داده ها نداشتند در حالی که در فاز دوم در متغیر های اجتماعی شدن (P<0/001) و فعالیت های زندگی روزمره (P<0/01) تاثیر آماری معناداری مشاهده گردید. نتیجه‌گیری: یافته های حاصل از این تحقیق حاکی از آن است که آموزش مهارت های تمرین با توپ همزمان با دریافت درمان رایج می تواند به بهبود رفتار تطابقی کودکان مبتلا به اوتیسم با عملکرد بالا کمک کرده و همچنین از شدت علایم اختلال اوتیسم بکاهد.
